# Natural history of non-functioning pituitary neuroendocrine tumours: impact of baseline characteristics and gender-specific clinical presentations

**DOI:** 10.1007/s11102-026-01669-7

**Published:** 2026-03-27

**Authors:** Alessandro Bavaresco, Giulia Bovo, Alessandro Mondin, Carla Scaroni, Giacomo Voltan, Pierluigi Mazzeo, Irene Tizianel, Filippo Ceccato, Mattia Barbot

**Affiliations:** 1 Department of Medicine-DIMED, University of Padova, Via Ospedale Civile 105, Padova, 35128 Italy; 2 Endocrinology Unit, University Hospital of Padova, Via Ospedale Civile 105, Padova, 35128 Italy

**Keywords:** Non-functioning pituitary neuroendocrine tumours, Gender, Active surveillance, Surgery

## Abstract

**Background:**

Non-functioning pituitary neuroendocrine tumours (NF-PitNETs) are the most common pituitary macroadenomas, with highly variable clinical behaviour. Although tumor growth and functional impairment are key considerations, the impact of patient sex on presentation and outcomes remains incompletely characterized.

**Objective:**

To investigate clinical, radiological and histopathological predictors of long-term outcomes in NF-PitNETs managed conservatively or surgically and to evaluate gender impact on initial presentation and tumour natural history.

**Methods:**

We retrospectively analyzed 170 patients with NF-PitNETs, including those under active surveillance and those treated surgically, with or without adjuvant therapies. Long-term clinical, endocrine and radiological follow-up was performed.

**Results:**

Compared with female patients, males exhibited larger tumour dimensions and a higher prevalence of both visual field defects and endocrine dysfunction. Women, particularly in the surveillance cohort, had smaller tumours, often detected earlier, likely due to reproductive-age clinical monitoring. Despite these baseline differences, progression rates were similar between genders. In patients under surveillance, five-year tumour growth risk was higher for macroadenomas than microadenomas (45.7% vs. 22.5%, *p* = 0.030), and baseline tumour volume and visual impairment predicted progression. In the surgical cohort, residual tumour increased progression risk, while a > 40% volume reduction was associated with visual improvement. The Trouillas clinicopathological classification was not predictive of outcomes. Adjuvant therapies, including stereotactic radiosurgery and temozolomide, stabilized disease in most treated cases.

**Conclusions:**

Gender significantly influences NF-PitNETs presentation, with males harbouring larger, more symptomatic tumours whereas females tended to have an earlier detection. However, long-term progression seems to be independent of gender. These findings pinpoint the importance of individualized approach combining anatomical, volumetric, and functional assessments to optimize outcomes.

## Introduction

Non-functioning pituitary neuroendocrine tumours (NF-PitNETs) account for approximately 22–54% of all pituitary neuroendocrine tumours [1–4]. They do not induce clinically relevant hormone overproduction except for stalk-related hyperprolactinemia [5, 6] and are the most common pituitary macroadenomas [6]. The reported prevalence ranges from 7 to 41.3 per 100,000 individuals [6], although it is probably underestimated due to asymptomatic or incidental presentation; indeed, recent population studies suggest an even greater incidence [7]. NF-PitNETs are typically diagnosed in mid-to-late adulthood, with some studies indicating a higher frequency of large symptomatic tumours in men [8, 9].

Despite recent WHO classifications enhancing the histopathological characterization of pituitary tumours through the incorporation of transcription factor expression [10–12], thus emphasizing the biological diversity within the NF-PitNETs group, clinical behaviour is still mostly defined by anatomical and radiological features rather than by immunohistochemical subtype.

Clinical manifestations are mainly attributable to mass effects, including headache, visual field defects caused by optic chiasm compression, cranial nerve deficits related to cavernous sinus invasion, hypopituitarism and less frequently, pituitary apoplexy [13–19]. Management strategies may vary between active surveillance and transsphenoidal surgery (TSS), depending on symptoms, tumour size, proximity to critical structures and its pattern of growth [20–26]. However, the optimal intensity and duration of endocrine and radiological follow-up remain uncertain, especially in asymptomatic patients, reflecting the lack of full knowledge of the long-term natural history of these lesions [27–29]. Therefore, there is an urgent need for more precise predictive factors incorporating clinical, radiological and anatomical features to better stratify the risk of tumour progression and functional impairment in individual patients with NF-PitNETs [30, 31].

In this context, we conducted a single-centre retrospective study including patients managed either conservatively or surgically, tracking the natural history of all cases as well as the type and number of treatments required for disease control. By evaluating radiological evolution, endocrine outcomes, and surgical results, together with key anatomical parameters such as Knosp grade, optic chiasm involvement, and a composite invasiveness score, our aim was to better characterise the natural history of these tumours.

## Materials and methods

### Radiological assessment

This single-centre retrospective cohort study was based on a review of clinical records and all available pituitary magnetic resonance imaging (MRI) scans. For each patient, tumour size at diagnosis and during follow-up was calculated using the ellipsoid formula (V = 4/3 π × A/2 × B/2 × C/2), allowing assessment of volumetric changes over time. Adenomas were classified as microadenomas (< 10 mm), macroadenomas (≥ 10 mm), or giant adenomas (≥ 40 mm). A dimensional increase was considered significant when tumour volume increased by ≥ 80 mm³ or when any diameter increased by ≥ 2 mm [18, 32]. 

Parasellar extension was graded using the Knosp classification, while the relationship between the tumour and the optic chiasm was categorised considering 0 for distant, 1 for abutting, 2 for compressing. A composite anatomical invasiveness score was derived by combining Knosp grade (0 for grades 0–2; 1 for grades 3–4) and chiasmal involvement (0 for distant; 1 for abut and compression), yielding a score of 0–2.

For surgically treated patients, a postoperative MRI within 6 months was mandatory, followed by scans at 12, 24, 60 months and at the most recent available follow-up. The same imaging schedule was applied to patients receiving stereotactic radiosurgery. For patients receiving temozolomide, the first MRI re-evaluation was performed after completion of the third chemotherapy cycle. In conservatively managed patients, MRI at diagnosis and subsequent scans at 6, 12, 24, 60 months and at last available follow-up were reviewed to assess tumour evolution.

### Hormonal evaluation

Pituitary function was evaluated at diagnosis and throughout follow-up according to clinical need. Central adrenal insufficiency was confirmed by dynamic testing (short Synacthen) when indicated. Hypogonadotropic hypogonadism was defined as low, or inappropriately normal levels of follicle-stimulating hormone (FSH) and luteinizing hormone (LH) coupled with a morning testosterone below the reference range in males or low oestradiol and oligo/amenorrhoea in women of reproductive age or as gonadotropins below the age reference in post-menopausal women; central hypothyroidism was defined as low, or inappropriately normal thyroid-stimulating hormone (TSH) paired with free thyroxine below the reference range. Growth hormone deficiency was not routinely assessed at diagnosis because of limited feasibility and clinical relevance within this setting. Postoperatively, the growth hormone–releasing hormone (GHRH) plus arginine stimulation test was performed in patients with symptoms suggestive of GH deficiency, after exclusion of other pituitary hormone deficiencies or once adequate hormonal replacement had been achieved.

Patients with visual symptoms or with tumours close to or compressing the optic chiasm underwent visual field assessment by an ophthalmologist.

In patients undergoing surgery, available histopathological analyses were reviewed, including immunohistochemistry for transcription factors and pituitary hormones, Ki-67 index, p53 expression, and mitotic count.

In patients undergoing surgery, available histopathological analyses were reviewed, including immunohistochemistry for pituitary hormones and transcription factors, Ki-67 index, p53 expression, and mitotic count. Tumours were classified according to the clinicopathological classification proposed by Trouillas et al. [33], which integrates proliferative markers and tumour invasiveness. Proliferative status was defined by the presence of at least one of the following criteria: Ki-67 index ≥ 3% (formalin-fixed specimens), mitotic count > 2 per 10 high-power fields (HPF), or p53 overexpression (> 10 strongly positive nuclei per 10 HPF). Based on proliferative status and evidence of tumour invasion, tumours were classified into grades 1a, 1b, 2a, and 2b according to the Trouillas classification.

### Statistical analysis

Statistical analysis included Shapiro–Wilk testing for normality. Continuous variables are expressed as median and interquartile range; comparisons were made using Student’s t-test or Mann–Whitney U-test as appropriate, categorical variables with chi-square or Fisher’s exact test. Kaplan–Meier curves and Log-Rank tests were used to assess progression-free survival. Predictors of progression were evaluated using Cox regression. Bivariate correlations were analysed using Spearman’s rank correlation. Statistical significance was set at *p* < 0.05. Data analysis was performed using SPSS version 24 software package for Windows (SPSS Inc., Version 24, Chicago, IL, USA).

## Results

### Baseline characteristics of the entire cohort

A total of 170 adults with NF-PitNETs were evaluated between 2009 and 2025. The cohort included 91 men (54%) and 79 women (46%), with a median age of 60.5 years (49–69); men were older (63 vs. 57 years). Most tumours were macroadenomas (126, 74.1%), followed by microadenomas (38, 22.4%) and giant adenomas (6, 3.5%). Median tumour volume was 2640 mm³ (452–5648).

Visual disturbances were present in 58 patients (34.1%), including 51 visual field defects and 7 other manifestations. Additional symptoms were reported in 50 patients, most commonly headache (*n* = 25), vertigo/syncope (*n* = 11), and nausea/vomiting (*n* = 7). Endocrine function was normal in 125 patients (73.5%), while 45 patients (26.5%) had one or more pituitary hormone deficiencies, Table 1. One patient with a microadenoma had AVP deficiency at diagnosis; however, this represented a congenital condition due to the absence of the posterior pituitary.

**Table 1:** Baseline demographic, radiological, clinical, and endocrine characteristics

Characteristic	Value
Total patients	170
Sex	91 M (54%); 79 F (46%)
Median age at diagnosis (years)	60.5 (49–69)
Radiology	
Microadenomas	38 (22.4%)
Macroadenomas	126 (74.1%)
Giant adenomas	6 (3.5%)
Visual impairment	58 (34.1%)
Visual field defects	51 (30%)
Other disturbances	7 (4.1%)
Other symptoms	50 (29.4%)
Headache	25 (14.7%)
Vertigo/syncope	11 (6.5%)
Nausea/vomiting	7 (4.1%)
Hormonal status	
No hormonal deficits	125 (73.5%)
Hormonal deficits	45 (26.5%)
Types of deficits	
Pan-hypopituitarism	17 (10%)
Pan-hypopituitarism + AVP deficiency	1 (0.6%)
Anterior hypopituitarism	26 (15%)
Central adrenal insufficiency	4 (2.4%)
Hypogonadism	6 (3.5%)
Central hypothyroidism	7 (4.2%)
Combined deficits	10 (5.9%
AVP deficiency	1 (0.6%)

## Patients managed with active surveillance

Ninety patients (52.9%) were initially managed with active surveillance. Of these, 85 remained under observation throughout follow-up, whereas 5 subsequently required surgical intervention. The duration of observation ranged from 6 to 120 months (mean 35.6 months).

At baseline, 52 patients (57.8%) had macroadenomas and 38 (42.2%) had microadenomas, with a median tumour volume of 565 mm³ (range 48–2323). Visual impairment was present in 9 patients (10%), while other symptoms were reported in 20 (22.2%). Conservative management was pursued in these patients with visual deficits due to advanced age, significant comorbidities or patient refusal of surgery. Normal pituitary function was observed in 74 patients (82.2%), whereas 16 patients had hormonal deficits, including panhypopituitarism (*n* = 6), anterior hypopituitarism (*n* = 9), and AVP deficiency (*n* = 1).

Radiological progression occurred in 25 patients (27.8%), while 65 (72.2%) showed stable disease. Baseline characteristics of patients with and without tumour growth during follow-up are shown in Table 2. Median tumour volume at last evaluation was 727 mm³ (IQR 48.1–2430).

**Table 2:** Baseline characteristics according to tumour growth during follow-up

Variable	Growth (*n* = 25)	No growth (*n* = 65)	*p*
Age (years)	66 (54–75)	63 (48–72)	0.41
Male sex	13 (52%)	24 (37%)	0.22
Macroadenoma	19 (76%)	33 (51%)	0.041
Tumour volume (mm³)	1287 (531–3810)	324 (45–1043)	0.004
Visual deficits	6 (24%)	3 (4.6%)	0.018
Hormonal deficits	6 (24%)	10 (15%)	0.34
Knosp ≥ 3	3 (12%)	4 (6%)	0.39
Chiasmal contact/compression	11 (44%)	11 (17%)	0.012

**Fig. 1:**
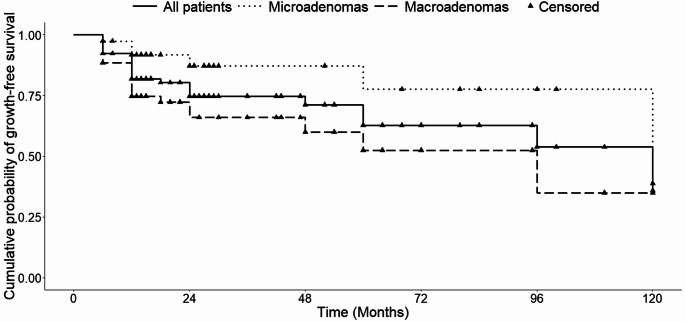
Cumulative probability of NF-PitNETs growth-free survival. Number of patients at risk: 90 (baseline), 83 (6 months), 71 (12 months), 40 (24 months), 15 (60 months), 2 (120 months)

Kaplan–Meier analysis showed a progressive decline in progression-free survival. Cumulative growth probability was 7.8% at 6 months, 18.2% at 12 months, 25.3% at 24 months, 37.2% at 60 months, and 64.1% at 120 months, (Fig. 1).

Macroadenomas exhibited a significantly higher risk of growth compared with microadenomas (log-rank *p* = 0.02).

On Cox analysis, baseline tumour volume (HR 1.27 per 1000 mm³; *p* = 0.004) and visual impairment (HR 3.63; *p* = 0.007) were identified as significant predictors of growth. Age and sex did not correlate to progression. A multivariable Cox regression including baseline visual impairment, tumour volume, age and sex was overall significant (score test *p* = 0.008), although none of the individual variables remained independently associated with tumour growth, Table 3.

Baseline hormonal deficits were not significantly associated with tumour growth on univariate Cox regression analysis (HR 0.54; *p* = 0.319). Growth was associated with the development of new hormonal deficits (20% vs. 4.6%; Fisher *p* = 0.035; OR 5.17), but not with new visual deficits (1 event only).

In univariate Cox regression analysis, suprasellar extension alone was associated with an increased risk of tumour growth, although the association did not reach statistical significance (HR 2.17, *p* = 0.096), suggesting a trend toward a higher risk of growth in patients with suprasellar extension.

As regards anatomical predictors, there was no correlation between Knosp grade and growth (*p* = 0.493). In contrast, Suprasellar extension with chiasmal contact correlated with higher growth probability (ρ = 0.328; *p* = 0.002), as did the combined anatomical score (ρ = 0.310; *p* = 0.003). Results were similar when growth was considered as percent volume increase (Knosp grade, *p* = 0,440; chiasmal contact ρ = 0.256; *p* = 0.015; combined score ρ = 0.328, *p* = 0.002).

**Table 3:** Predictors of tumour growth on Cox regression analysis

Variable	Univariable HR	*p*	Multivariable HR	*p*
Tumour volume	1.27	0.004	1.00	0.107
Visual impairment	3.61	0.007	2.42	0.142
Age	1.01	0.533	0.99	0.630
Sex	1.50	0.317	1.28	0.601

## Patients undergoing surgery

Eighty-five patients underwent surgery, of whom 80 in the first year following diagnosis and 5 after initial active surveillance. Fifteen required a second TSS and 4 a third procedure. Stereotactic radiosurgery was performed in eight patients, and temozolomide was administered to 3 cases.

At baseline, 78 patients had macroadenomas and 7 giant adenomas, with a median preoperative volume of 5422 mm³ (IQR 3177–10441). Six months postoperatively, 24 patients had no radiological residual of whom only one of experienced recurrence.

**Fig. 2:**
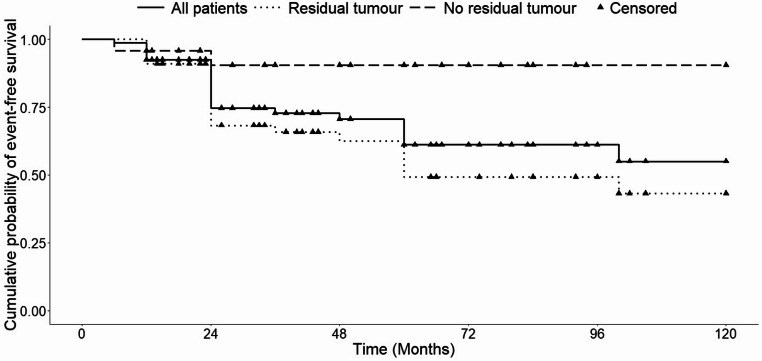
Cumulative probability of event-free survival after surgery

Patients with postoperative tumour remnant had significantly lower event-free survival (defined as growth or need for additional treatment) compared with those without residual disease (log-rank *p* = 0.028), (Fig. 2).

Univariate Cox regression suggested a trend toward significance for residual volume (HR 1.06 per 1000 mm³; *p* = 0.058). No significant associations were observed for age or sex. Multivariate analysis did not identify any independent predictors of event-free survival.

There was a marked increase in new hormonal deficits during the early postoperative period (0–6 months), with an incidence rate of 65.9 events per 100 person-years (28 events among 85 patients at risk). The incidence decreased sharply thereafter, with rates of 5.1, 4.6, and 0.9 events per 100 person-years during the 6–12, 12–24, and 24–60 month intervals, respectively. Beyond 60 months, the incidence remained low at 1.8 events per 100 person-years.

Among 50 patients with preoperative visual field defects, 23 still showed deficits at 6–12 months Improvement at 6 months was significantly associated with a > 40% reduction in tumour volume (Pearson χ², *p* = 0.010). Only one patient developed new visual field impairment during long-term follow-up.

Knosp grade was negatively correlated with postoperative volume reduction (ρ = − 0.521; *p* < 0.001). Chiasmal compression showed a weaker but significant association (ρ = − 0.250; *p* = 0.021). Combined anatomical score also correlated with volume reduction (ρ = − 0.283; *p* = 0.009).

Mann–Whitney tests confirmed that patients with postoperative residual tumour had significantly higher Knosp grades and greater chiasmal proximity compared with those without residual disease (*p* = 0,0001 and *p* = 0,04, respectively). Among patients with residual tumour, only chiasmal proximity, not Knosp grade nor the combined anatomical score, correlated with subsequent residual growth or need for second-line treatment (ρ = 0.263; *p* = 0.050).

### Immunohistochemical evaluation

The Trouillas clinicopathological classification was available for 40 patients with residual tumour, Table 4. When analysed according to the four original categories (1a, 1b, 2a, 2b), no significant association was observed between Trouillas grade and tumour progression or need for additional treatment (χ² = 2.145, *p* = 0.543). To further explore the components of the classification, additional analyses were performed by grouping tumours according to invasiveness (grades 1a–1b vs. 2a–2b) and proliferative status (grades 1a-2a vs. 1b-2b). No significant difference in the rate of progression or additional treatment was found between non-invasive and invasive tumours (44.0% vs. 46.7%; χ² = 0.027, *p* = 0.870; Fisher’s exact *p* = 1.000). Similarly, proliferative tumours showed a numerically higher rate of events compared with non-proliferative lesions (57.1% vs. 42.4%), but the difference did not reach statistical significance (χ² = 0.505, *p* = 0.477; Fisher’s exact *p* = 0.680.

**Table 4:** Distribution of Trouillas grades in patients with residual tumour

Trouillas grade	No event	Event	Total	Event rate (%)
1a	13	8	21	38.1
1b	1	3	4	75
2a	6	6	12	50
2b	2	1	3	33.3
Total	22	18	40	45

Immunohistochemically, operated NF-PitNETs were classified as in Table 5. Silent gonadotroph tumours represented the most common subtype (39/64 with available immunohistochemistry; 61%), consistent with their predominance in published series. Among patients with silent gonadotroph tumours and postoperative residual disease, proliferative activity was not associated with residual growth or retreatment (Fisher *p* = 1).

**Table 5:** Immunohistochemical classification of operated NF-PitNETs

Tumour subtype	*n*	Details
Silent gonadotroph PitNETs	39	24 SF1-positive; 6 FSH/LH-negative; 13 GATA3-positive; 15 LH/FSH-positive without transcription factor evaluation
Silent corticotroph	7	3 ACTH and TPIT positive; 3 ACTH-positive; 1 TPIT-positive
Silent lactotroph	2	PRL-positive; 1 also PIT1-positive
Null-cell adenoma	1	–
PIT1-positive plurihormonal	2	One GH/PRL-positive; one GH/PRL/TSH-positive
Unusual plurihormonal	1	ACTH + FSH positivity; transcription factors not assessed
Hormone-negative tumours	11	Transcription factors not evaluated
Triple positive (SF1, TPIT, PIT-1)	1	Likely normal adenohypophyseal tissue

### Second- and third-line treatments

**Fig. 3:**
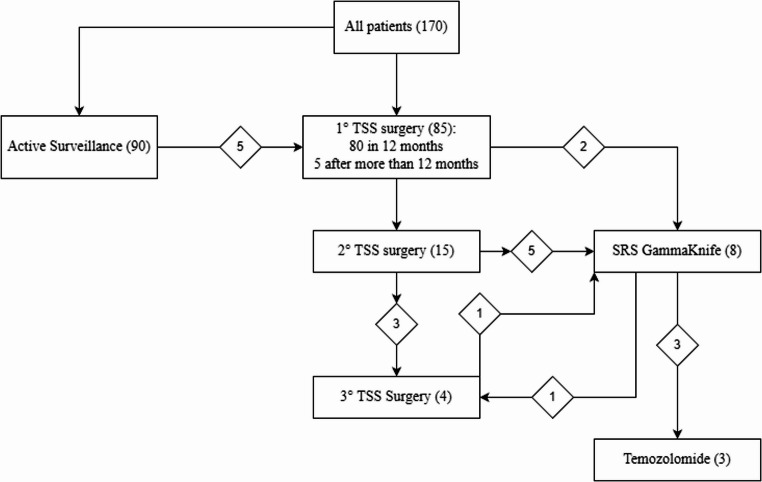
Flowchart of therapeutic interventions undertaken in the entire cohort (TSS: transsphenoidal surgery; SRS stereotactic radiosurgery)

Overall, 15 patients required a second surgery (median 35 months after the first), achieving significantly smaller volume reduction compared with the first TSS (*p* = 0.017), and only one complete resection. Four patients required a third surgery, with one complete resection achieved; two developed new hormone deficiencies. Eight patients received stereotactic radiosurgery (GammaKnife), with only one progressing and requiring temozolomide; one developed new hormonal deficits. An overview of the therapeutic interventions performed in the entire cohort is provided in Fig. 3. Three patients received temozolomide (after 1–3 surgeries and in combination with radiosurgery in two cases), with only one experiencing progression but without visual or endocrine worsening. Histological data across reoperations showed predominantly silent gonadotroph tumours, with no significant association was found between proliferative markers (Ki-67 ≥ 3%, p53, mitotic count) and tumour growth or need for treatment escalation.

### Sex-based comparisons in whole cohort

Men had significantly larger tumours (4089 vs. 805 mm³; *p* < 0.001) and were more likely to present visual impairment (38.5% vs. 20.3%; *p* = 0.008), hormonal deficits (40.7% vs. 10.1%; *p* < 0.001) and to undergo TSS (62.6% vs. 35.4%; *p* < 0.001), Table 6. Age did not differ significantly.

**Table 6:** Comparison between males and females at diagnosis

	Men (*n* = 91)	Women (*n* = 79)	*p*-value
Median age (years)	63 (52–69)	57 (47–67)	0.088
Median tumour volume (mm³)	4089 (1657–7322)	805 (52–3339)	< 0.001
Visual deficits (%)	38.5	20.3	0.008
Hormonal deficits (%)	40.7	10.1	< 0.001
Surgery (%)	62.6	35.4	< 0.001

### Gender-based comparisons in patients in active surveillance

Males in active surveillance had significantly larger tumours (1600 vs. 226 mm³; *p* = 0.001) and more baseline hormonal deficits (35.1% vs. 9.4%; *p* = 0.006). Growth occurred in 35.1% of men vs. 20.8% of women (ns), Table 7.

**Table 7:** Sex differences among patients under active surveillance

Variable	Men (*n* = 37)	Women (*n* = 53)	*p*-value
Median age (years)	67 (53–75)	55 (44–71)	0.014
Tumour volume t0 (mm³)	1600 (531–4397)	226 (31–888)	0.001
Hormonal deficits t0 (%)	35.1	9.4	0.006
Visual deficits t0 (%)	13.5	7.5	0.545
Tumour volume tlast (mm³)	1789 (619–4823)	256 (25–1385)	0.001
Growth during follow-up (%)	35.1	20.8	0.129
New hormonal deficits (%)	13.5	5.7	0.266

### Gender-based comparisons in surgically treated patients

Among the 85 surgically treated patients, the two genders had similar baseline age. Although preoperative tumour volume was larger in men, the difference was not statistically significant. Radical resection was more frequent in women (42.9% vs. 21.1%; *p* = 0.036).

Men developed significantly more new hormonal deficits during follow-up (47.4% vs. 25%; *p* = 0.048) and had higher persistence of visual field deficits at 6 months (36.8% vs. 7.1%; *p* = 0.004), Table 8.

**Table 8:** Gender differences in surgically treated patients

Median age (years)	Men (*n* = 57)	Women (*n* = 28)	*p*-value
	61 (51–67)	61 (51.3–67)	0.899
Visual deficits t0 (%)	60.0	57.0	0.825
Tumour volume t0 (mm³)	5648 (3321–10802)	4241 (1992–9991)	0.143
Tumour volume t1 (mm³)	1903 (471–5253)	2793 (461–4676)	0.780
Radical resection (%)	21.1	42.9	0.036
New hormonal deficits (%)	47.4	25.0	0.048
Visual field deficits t1 (%)	36.8	7.1	0.004

## Discussion

In this large, long-term cohort of patients with NF-PitNETs, we analysed the natural history of tumours managed with active surveillance and the outcomes of those undergoing TSS and multimodal treatment. The results highlight the marked biological and clinical heterogeneity of NF-PitNETs and reveal some prognostic determinants which may guide individualized management strategies.

Among the 90 patients managed with active surveillance, radiological progression occurred in 27.8%, consistent with historical series reporting growth in approximately 25–50% of untreated NF-PitNETs over 4–6 years of follow-up [9, 28, 34, 35]. The cumulative probability of progression steadily increased over time, reaching 37.2% at 5 years and 64.1% at 10 years, consistent with the concept that NF-PitNETs exhibit slow but continuous growth in a substantial subset of patients.

A clinically significant difference in growth risk was observed between macroadenomas and microadenomas (47.1% vs. 22.5% at 60 months), similar to that reported by Karavitaki and colleagues [34], with faster growth in larger tumours. Baseline tumour volume was a significant predictor of progression in univariate Cox analysis, confirming the predictive role of initial size reported in previous observational cohorts [36]. Age and sex had no impact on natural history, consistent with previous reports showing that demographic variables are not strong predictors of progression risk [34, 36]. Similarly, baseline pituitary hormone deficits were not associated with a higher risk pf tumour growth in our cohort, suggesting that endocrine dysfunction at diagnosis likely reflects the mass effect of larger tumours rather than intrinsic tumour aggressiveness.

Visual impairment at baseline also correlated with progression risk in univariate analysis. Although this association lost significance in multivariable modelling, its presence suggests that tumours large enough or positioned such that they compress the optic chiasm may possess features associated with increased biological activity, as suggested by Fountas et al. [9], who demonstrated similar associations in conservatively managed NF-PitNETs. This observation is consistent with our findings since lesions that abut or compress the optic chiasm, were also those with the highest probability of growth.

Conversely, cavernous sinus invasion assessed by Knosp grade, was not associated with tumour progression in our cohort. In contrast, the combined anatomical invasiveness score correlated with growth. These findings suggest that vertical extension into the suprasellar cistern may be more strongly associated with the risk of tumour growth than parasellar invasion alone.

An important functional outcome was the development of new pituitary hormone deficits, which occurred significantly more frequently in patients whose tumours grew during follow-up (20% vs. 4.6%). This relation emphasizes the importance of an increasing mass effect in the genesis of pituitary dysfunction, in line with prior studies where progressive growth proved a major risk factor for new endocrinopathies [9, 37]. Conversely, the worsening of pituitary function is rarely observed for stable lesions. These data confirm the necessity for routine endocrine evaluation in patients with documented radiological progression while suggesting that in stable lesions, hormonal monitoring can be tailored to clinical context.

Most of the 85 patients submitted to TSS were operated within the first year of diagnosis. For these patients, postoperative outcomes were strongly influenced by the presence of residual tumour. Complete resection resulted in excellent long-term disease control, with only one documented recurrence; this confirms results of previous studies indicating postoperative residue as the most powerful predictor of future treatment needs [18, 38]. Patients with incomplete resections had a significantly lower event-free survival, with even a trend suggesting that higher residual volume may predict an earlier progression.

The early postoperative period was associated with a very high incidence of new hormone deficits (65.9 events per 100 person-years during the first six months), thereafter, rates markedly declined reflecting a major contribution of surgical manipulation rather than tumour progression on pituitary disfunction. The incidence of new postoperative deficits in our cohort was higher compared to that reported by Fountas et al. [9] but similar to that described by Zhang et al. [39]. The absence of postoperative residue did not protect against new deficits, further reinforcing that hypopituitarism largely reflects immediate surgical impact. There was no evidence of any tumour growth among those receiving GH replacement, consistent with the lack of impact on recurrence risk demonstrated by van Varsseveld et al. in NF-PitNETs [40], confirming the safety of this therapy.

Improvement in visual fields at 6–12 months was observed in approximately half of patients who had baseline deficits, and a reduction in tumour volume of more than 40% was significantly associated with recovery. These findings suggest that substantial decompression, rather than complete resection, is a key determinant of functional improvement. Similar results were reported by Dekkers et al. [41], who reported progressive visual recovery during the first postoperative year independently of complete tumour removal.

Our findings highlight the impact of anatomical complexity on surgical outcomes. As expected, cavernous sinus invasion, reflected by higher Knosp grades, limited the extent of tumour debulking, reinforcing the well-recognized challenge of achieving complete resection in these cases. Interestingly, chiasmal compression and the combined anatomical score showed only a weaker association with reduced debulking and postoperative residue; among patients with residual tumour, chiasmal proximity appeared to predict subsequent growth or the need for additional intervention. This suggests that suprasellar extension may capture aspects of biological aggressiveness not fully reflected by parasellar invasion alone, emphasizing the value of accurate anatomical assessment in risk stratification and surgical planning.

Immunohistochemical analysis confirmed silent gonadotroph tumours as the predominant NF-PitNET subtype, consistent with large surgical series and supporting the dominance of the SF1-lineage in these lesions [42, 43].

No significant association emerged between the Trouillas clinicopathological classification and tumour progression or the need for additional treatment in our cohort. Previous studies have suggested that the invasive and proliferative components of this classification may identify pituitary neuroendocrine tumours with a higher risk of recurrence or progression [44–46]. The lack of association observed in our series may be related to the relatively limited number of patients with available Trouillas grading, particularly within the proliferative subgroups, which may have reduced the statistical power to detect potential differences.

Second TSS showed the practical challenges posed by remnants in anatomically restricted areas. Smaller reductions after second surgeries likely reflect both more complex tumour geometry and possibly more aggressive tumour biology. Adjuvant stereotactic radiosurgery provided disease stability in the majority of patients, consistent with reported 5–10-year control rates of 85–95% [47]. Only one SRS-treated patient progressed, and new endocrinopathies were infrequent. Temozolomide (TMZ) with minimal new endocrinopathies. Temozolomide, used selectively in refractory or aggressive tumours, stabilized disease in two of three cases, echoing the reported response rate of approximately 40% [48] and reinforcing its role as a therapeutic option in highly resistant pituitary tumours [49].

Gender-related differences were evident in tumour presentation and outcomes. Male patients presented with larger tumours and consequently higher rates of visual and endocrine dysfunction, supporting prior observations that men often present with more pronounced mass-effect symptoms due to later detection [8]. In contrast, women in the active surveillance group were younger and had smaller tumours, likely reflecting earlier or incidental detection during the reproductive years, often prompted by menstrual irregularities or mild hyperprolactinemia.

Among the surgical cohort, females achieved complete resection more frequently and experienced fewer postoperative hormonal deficits and lower rates of visual impairment. These differences likely stem from males harbouring larger tumours closer to the optic chiasm-factors known to complicate surgical removal and increase the risk of pituitary injury [39]. Nevertheless, consistent with previous reports, TSS provided meaningful visual improvement in both sexes, particularly in patients with severe preoperative chiasmal compression [41].

### Overall implications

Taken together, these findings highlight several important points. Firstly, NF-PitNETs require a personalized risk assessment that incorporates not only tumour size and clinical presentation but also anatomical features, such as suprasellar extension and optic chiasm abutment, which in our cohort were stronger predictors of growth than cavernous sinus invasion. These radiological markers, combined with early postoperative imaging, provide a more detailed and nuanced approach to understanding tumour behaviour. Secondly, despite the limited prognostic value of proliferative markers and the Trouillas classification in our cohort, anatomical and volumetric features remained key determinants for clinical surveillance and surgical planning. Multimodal therapy, which may include radiosurgery and TMZ, should be considered for not fully resectable tumours with features of biologically aggressiveness.

Finally, gender-related differences appear driven primarily by tumour size at diagnosis rather than intrinsic biological variation, underscoring the importance of timely detection to reduce visual and endocrine morbidity. Upcoming efforts should be devoted to establishing integrated predictive models that combine clinical, radiological and molecular features, in order to facilitate long-term risk stratification and personalize management ​‍​‌‍​‍‌​‍​‌‍​‍‌strategies.

## Data Availability

The data supporting the findings of this study are not publicly available due to patient privacy and ethical restrictions but are available from the corresponding author upon reasonable request.
